# Current clinical practice for Parkinson’s disease among Chinese physicians, general neurologists and movement disorders specialists: a national survey

**DOI:** 10.1186/1471-2377-12-155

**Published:** 2012-12-07

**Authors:** Wei Chen, Shuai Chen, Qin Xiao, Gang Wang, Sheng-Di Chen

**Affiliations:** 1 Department of Neurology & Institute of Neurology, Ruijin Hospital affiliated to Shanghai Jiao Tong University School of Medicine, Shanghai, 200025, China

**Keywords:** Parkinson’s disease, National survey, Clinical practice guideline, Medication

## Abstract

**Background:**

To explore current status and choices regarding diagnosis and treatment of Parkinson’s disease (PD) among physicians, general neurologists and movement disorders specialists in China via a national survey.

**Methods:**

The cross-sectional questionnaire-based survey was conducted from November, 2010 to July, 2011. Six hundreds and twelve doctors from different cities in China were recruited for this study.

**Results:**

68.6% (*n*=420) and 23.9% (*n*=146) of doctors have read the national and international guidelines, respectively. There was a larger proportion of movement disorders specialists reading the guidelines, in contrast to physicians and general neurologists (*P<0.001*). Up to 76.4% (*n*=465) and 81.8% (*n*=498) of doctors would choose standard oral levodopa test and conventional MRI(with T1 and T2), respectively; Whereas susceptibility weighed imaging(SWI)(16.1%; *n*=98), transcranial sonography (TCS) (1.8%; *n*=11) and functional neuroimaging test, such as single photon emission computed tomography(SPECT) (10.2%; *n*=62) and positron emission tomography(PET)(13.3%; *n*=81) were less used for suspected patients with PD in clinical practice. Doctors at different levels or from different hospitals and cities would choose different medication for motor complications and non-motor symptoms of patients with PD, in addition to initial drug selection for newly diagnosed PD. Doctors who had read the guidelines had significantly better knowledge of medication selections for PD under specific circumstances.

**Conclusions:**

Compared with commonly employed standard oral levodopa test and conventional MRI, SWI complements MRI, TCS and functional neuroimaging were less performed for diagnosis of PD in clinical practice in China. The choices of diagnostic methods and therapeutic strategy of PD vary among physicians, general neurologists and movement disorders specialists. Guideline awareness is markedly beneficial to reasonable PD medications strategy in China.

## Background

Recently, epidemiological investigations indicated that there were approximately at least two million patients with Parkinson’s disease (PD) in China, accounting for 2/5 of the whole PD patients in the world [1]. Over the past three decades, significant progresses have been achieved on the diagnosis and treatment of PD in China. So far, the academic Chinese guidelines (First version(2006) and updated version (2009)) on the treatment of PD have been published by Chinese Parkinson’s Disease & Movement Disorders Society (CPDMDS) in recent years [2,3], respectively, soon after the dissemination of the counterparts from American Academy of Neurology (AAN) [4-7], European Federation of Neurological Societies (EFNS) and National Institute for Health and Clinical Excellence (NICE) in the United Kingdom [8]. It seems that the gap of diagnosis and management of PD patients is narrowing between China and Western Countries. However, the practical status of choices and opinions regarding diagnosis and treatment of PD among Chinese doctors is unknown unfortunately [9]. It is believed as a key influential factor to determine the actual benefit for patients [10-13].

In China, in addition to movement disorders specialists, both general neurologists and physicians are caring for PD patients. Each province has one capital city (such as Nanjing city as capital city of Jiangsu Province and Guangzhou city as capital of Guangdong Province) with integrated financial and economic centers. More medical resources were distributed in a few tertiary class hospitals in capital cities with over 1000 beds, followed by secondary class hospitals mostly located in the county with over 400 beds and primary health service centers [14,15]. The movement disorders clinics and associated centers only exist in part of tertiary hospitals, and the resources could not satisfy the need of most patients with PD. In many cases, both general neurologists and physicians are taking the responsibility for diagnosis and treatment of PD patients. Therefore, the opinions from physicians, general neurologists and movement disorders specialists in the clinical practice for PD will be diverse, which will have significant influence on the actual benefits of patients with PD.

Therein, we designed a national survey to investigate the opinions regarding diagnostic approaches for suspected PD, medications selection strategy for newly diagnosed PD, and motor complications & non-motor symptoms (NMSs) for follow-up patients among doctors at different levels.

## Methods

### Survey setting and sample

The study was sponsored by Chinese Parkinson’s Disease & Movement Disorders Society, Neurology Branch of Chinese Medical Association. From November, 2010 to July, 2011, questionnaires were randomly distributed to doctors attending National Neurological Congress, several Provincial Conferences or CME courses of PD held in Beijing, Shanghai, Chongqing, Guangzhou, Zhengzhou, Nanning and Hangzhou, respectively. In this survey, we classified the congress participants into three categories by their specialties and sub-specialties: A movement disorders specialist is a neurologist who has taken additional training in the subspecialty in neurology called movement disorders (as compared to other subspecialties in neurology) and regularly attends the movement disorders clinics; A neurologist who specializes in general neurology rather than movement disorders, is classified as general neurologists; Physicians are the doctors who did not work in neurology department, including general physicians and internal medicine specialists.While family physicians were not included in this survey. All of the abovementioned three categories of doctors had qualified medical licenses issued by the Chinese Public Health Administration.

**Figure 1:**
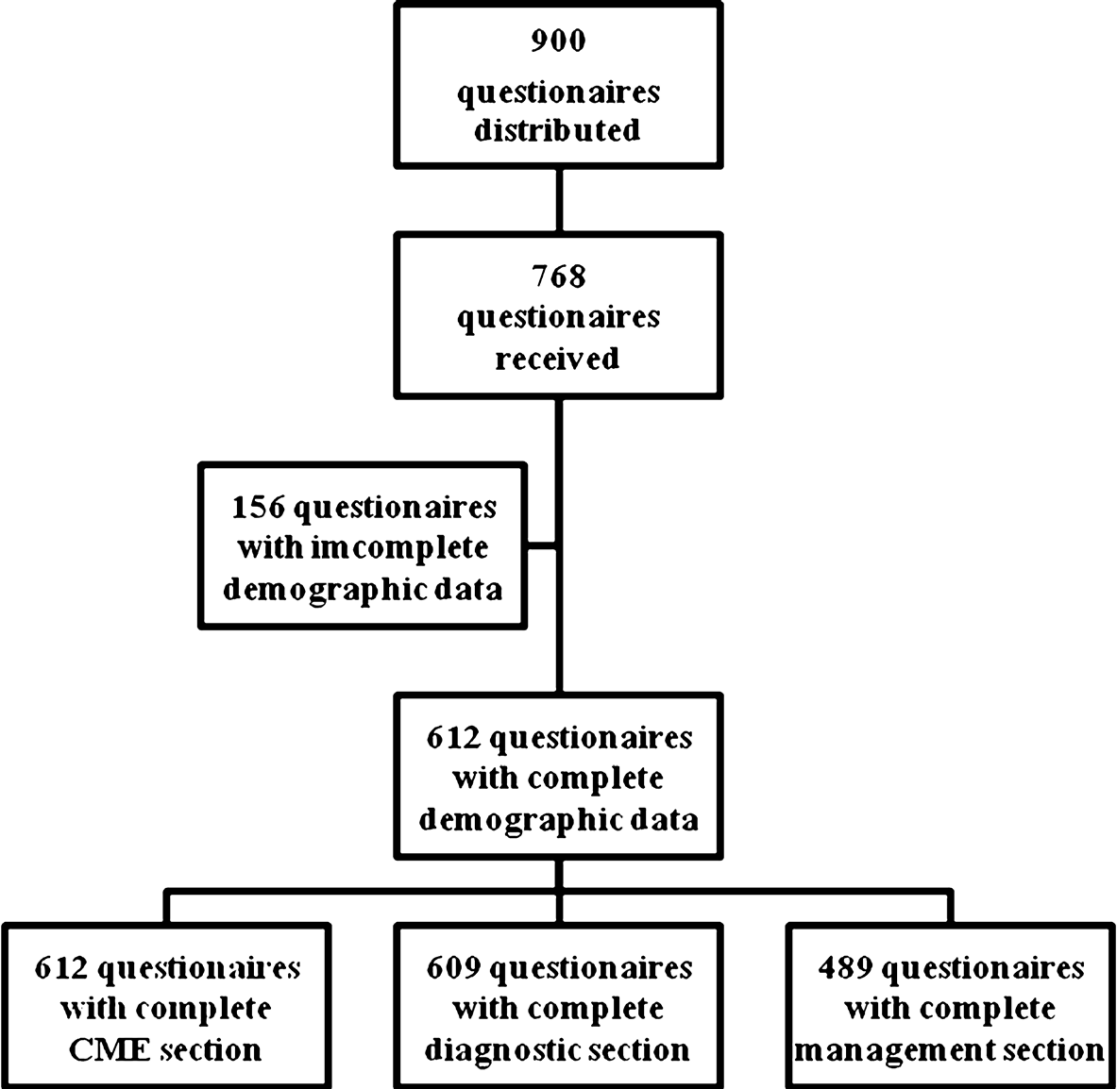
Screening strategy for questionnaires through the survey.

Finally, a total of 900 questionnaires were distributed, 768 questionnaires were returned (response rate 85.3%), and 612 (effective rate 79.7%) questionnaires with complete demographic data were available for further evaluation (Figure 1). Of the 612 participants, 328(53.6%) were male. 248(40.5%) were below 35 and 352(57.5%) were 35–60 years old. 406(66.3%) responders came from tertiary hospitals, only 181(29.6%) and 25(4.1%) came from secondary hospitals and private clinics. Majority of the participants were general neurologists (70.8%, *n*=433) rather than physicians (14.7%, *n*=90) and movement disorder specialists (14.5%, *n*=89).

### Questionnaire design

The questionnaire (Additional file 1) was designed by three of the authors (Dr. Gang Wang, Dr. Wei Chen and Dr. Sheng-Di Chen) with approval from the Research Ethics Committee, Ruijin Hospital affiliated to Shanghai Jiao Tong University School of Medicine, Shanghai, China. It covered three sections: (1) Continuing Medical Educations(CME) & research experiences for PD: consisting of guideline reading, specialty information source, CME activity frequency, PD patients to provide consultation service monthly on average, research papers published, etc.; (2) Initial diagnostic approaches for PD patients: including choices of a standard oral levodopa test, conventional magnetic resonance imaging (MRI), susceptibility weighed imaging (SWI), transcranial sonography (TCS), single-photon emission computed tomography (SPECT) and positron emission tomography (PET), in addition to assessment of putative pre-motor symptoms of PD; (3) Strategy on PD management: comprising of initial medications choices for newly diagnosed PD, how to deal with wearing-off phenomenon, peak-dose dyskinesia, and some common non-motor symptoms, including psychosis, dementia, depression and restless legs syndrome. The response categories were in multiple-choice format for easily completing. It took about 20–30 min for finishing.

### Statistical analysis

Statistical analysis was performed with SPSS, with student’s *t* test for comparing group means and chi-squared analysis for comparing proportions. The significance level was set at *P*<0.05.

## Results

### CME & research experiences for PD

Of the 612 participants, 68.6% (*n*=420) and 23.9% (*n*=146) have read the national and international guidelines, respectively. There was a larger proportion of movement disorders specialists (75/89, 84.3%) reading the national guideline, in contrast to general neurologists (302/433, 69.7%) and physicians (43/90, 47.8%) (*P<0.001*). The situation was similar for the international guidelines reading (51.7% for movement disorders specialists VS. 19.6% for general neurologists VS. 16.7% for physicians; *P<0.001*). There were 72.9% (*n*=446) of responders caring for below 10 PD patients each month; 17.8% (*n*=109) 10–30, and 5.4% (*n*=33) above 30 PD patients monthly. However, 3.8% (*n*=23) of those have never diagnosed and treated PD patients before, most of whom are from secondary hospitals or private clinics. There were more movement disorders specialists (48/89, 53.9%) caring for more than 10 PD patients each month, than the general neurologists (90/433, 20.8%) and physicians (4/90, 4.4%) (*P*<0.001).

From the survey, information with respect to the current advances in PD was most frequently obtained from lectures of movement disorders specialists (68.0%; *n*=416), followed by professional journals (59.8%; *n*=366), movement disorders textbooks (57.8%; *n*=354), guidelines (50.8%; *n*=311) and information from the pharmaceutical sales representatives (14.9%; *n*=91). Regarding CME activity frequency for PD, 54.2% (*n*=332) of participants attended once or twice every year. Only 18.8% (*n*=115) took part in CME activities more than 3 times each year. 26.1% (*n*=160) never attended any programs before, which included 48.9% (44/90) physicians, 25.1% (109/433) general neurologists, and 7.9%(7/89) movement disorders specialists. With respect to papers published for PD, up to 80.6% (*n*=493) participants had never published any article. Only 17.1% (*n*=105) and 9.5% (*n*=58) had published papers for clinical studies and basic researches on PD, respectively. There were 101 (16.5%) responders undertaking basic research of PD at the same time of regular clinical works at present.

### Initial diagnostic approaches for PD

Most participants, 76.4% (*n*=465), would adopt standard oral levodopa test as differential diagnosis method for patients with suspected PD. Conventional cranial MRI with T1 and T2 weighted images were performed by 81.8% (*n*=498) of those surveyed for diagnosis and differential diagnosis for PD. A small proportion of the responders would take SWI (16.1%; *n*=98), TCS (1.8%; *n*=11) and functional neuroimaging, such as SPECT (10.2%; *n*=62) and PET (13.3%; *n*=81) for PD diagnosis.

As putative pre-motor symptoms of PD, hyposmia, REM sleep behavior disorder(RBD), constipation, anxiety & depression were separately recognized as regular assessment items in the clinic by 42.5% (*n*=259), 43.5% (*n*=265), 36.3% (*n*=221) and 41.7% (*n*=254) of participants. There were significant differences regarding selection of diagnostic methods and putative pre-motor symptoms including hyposmia, RBD and constipation across the three doctor categories. Movement disorders specialists had a relatively higher cognition for related diagnostic methods (Table 1).

**Table 1:** Opinions regarding selection of diagnostic methods and putative pre-motor symptoms among doctors at different levels

**Items**	**Total (*n*=609)**	**Physicians (*n*=91)**	**General neurologists (*n*=429)**	**Movement disorders specialists (*n*=89)**	***P*value**
Levodopa test	465(76.4%)	49(53.8%)	340(79.3%)	76(85.4%)	0.000
Neuroimaging					
Conventional MRI	498(81.8%)	64(70.3%)	356(83.0%)	78(87.6%)	0.005
SWI	98(16.1%)	7(7.7%)	71(16.6%)	20(22.5%)	0.023
TCS	11(1.8%)	0(0%)	1(0.2%)	10(11.2%)	0.000
SPECT	62(10.2%)	9(9.9%)	34(7.9%)	19(21.3%)	0.001
PET	81(13.3%)	9(9.9 %)	47(11.0%)	25(28.1%)	0.000
Pre-motor symptoms					
Hyposmia	259(42.5%)	15(16.5%)	191(44.5%)	53(59.6%)	0.000
RBD	265(43.5%)	33(36.3%)	181(42.2%)	51(57.3%)	0.010
Constipation	221(36.3%)	21(23.1%)	157(36.6%)	43(48.3%)	0.002
Anxiety & depression	254(41.7%)	28(30.8%)	186(43.4%)	40(44.9%)	0.069

### Strategy on PD management

#### Initial medications for newly diagnosed PD

With regard to initial medications for PD patients aged below 65 years without cognitive impairments (CI), dopamine agonists were employed by 42.9% (*n*=210) of 489 participants, followed by levodopa (33.5%) and MAO-B inhibitors (12.7%). Meanwhile, levodopa was used by 56.6% (*n*=277) of those surveyed for PD patients aged above 65 years or with CI (Table 2).

**Table 2:** Drug selection strategy for PD patients under specific circumstances among doctors at different levels

**Items**	**Total (*n*=489)**	**Physicians (*n*=67)**	**General neurologists (*n*=344)**	**Movement disorders specialists (*n*=78)**	***P*value**
Age < 65 years without cognitive impairment					
Levodopa	164(33.5%)	28(41.8%)	114(33.1%)	22(28.2%)	0.216
Dopamine agonists	210(42.9%)	13(19.4%)	144(41.9%)	53(67.9%)	0.000
MAO-B inhibitors	62(12.7%)	8(11.9%)	37(10.8%)	17(21.8%)	0.030
Age > 65 years or with cognitive impairment					
Levodopa	277(56.6%)	30(44.8%)	192(55.8%)	55(70.5%)	0.007
Dopamine agonists	114(23.3%)	8(11.9%)	80(23.3%)	26(33.3%)	0.01
MAO-B inhibitors	41(8.4%)	7(10.4%)	26(7.6%)	8(10.3%)	0.597
Wearing-off phenomenon					
Add levodopa frequency	187(38.2%)	14(20.9%)	131(38.1%)	42(53.8%)	0.000
Switch to CR levodopa	236(48.3%)	15(22.4%)	173(50.3%)	48(61.5%)	0.000
Add COMT inhibitors or MAO-B inhibitors	203(41.5%)	21(31.3%)	136(39.5%)	46(59.0%)	0.001
Add dopamine agonists	196(40.1%)	15(22.4%)	143(41.6%)	38(48.7%)	0.003
Peak-dose dyskinesia					
Reduce levodopa dose, add its frequency	218(44.6%)	24(35.8%)	149(43.3%)	45(57.7%)	0.021
Reduce levodopa dose, add dopamine agonists	215(44.0%)	15(22.4%)	151(43.9%)	49(62.8%)	0.000
Reduce levodopa dose, add COMT inhibitors	145(29.7%)	15(22.4%)	97(28.2%)	33(42.3%)	0.018
Add amantadine	71(14.5%)	8(11.9%)	38(11.0%)	25(32.1%)	0.000
PD with psychosis					
Clozapine	112(22.9%)	11(16.4%)	67(19.5%)	34(43.6%)	0.000
Olanzapine	224(45.8%)	27(40.3%)	162(47.1%)	35(44.9%)	0.584
Quetiapine	106(21.7%)	9(13.4%)	68(19.8%)	29(37.2%)	0.001
PD with dementia					
Huperzine A	124(25.4%)	8(11.9%)	101(29.3%)	15(19.2%)	0.004
Donepezil	263(53.8%)	30(44.8%)	178(51.7%)	55(70.5%)	0.003
Rivastigmine	82(16.8%)	10(14.9%)	50(14.5%)	22(28.2%)	0.013
Memantine	185(37.8%)	15(22.4%)	126(36.6%)	44(56.4%)	0.000
PD with depression					
Tricyclic antidepressants	101(20.7%)	13(19.4%)	79(23.0%)	9(11.5%)	0.077
SSRIs	292(59.7%)	31(46.3%)	200(58.1%)	61(78.2%)	0.000
Pramipexole	185(37.8%)	16(23.9%)	125(36.3%)	44(56.4%)	0.000
PD with RLS					
Levodopa	123(25.2%)	11(16.4%)	86(25.0%)	26(33.3%)	0.064
Dopamine agonists	149(30.5%)	15(22.4%)	90(26.2%)	44(56.4%)	0.000
Benzodiazepines	140(28.6%)	15(22.4%)	108(31.4%)	17(21.8%)	0.114

#### Motor complications treatment

For patients with wearing-off phenomenon, the most common therapeutic strategy was a switch from standard levodopa to CR levodopa (48.3%; *n*=236) among those surveyed. Additionally, enhancement of levodopa frequency (38.2%; *n*=187) and usage of some additives, such as dopamine agonists (40.1%, *n*=196) and COMT or MAO-B inhibitors (41.5%, *n*=203) were also regularly employed; 44.6% (*n*=218) of participants would reduce levodopa dose and add its frequency for patients with peak-dose dyskinesia, whereas only 14.5% (*n*=71) of those surveyed would choose amantadine for such condition (Table 2).

#### Non-motor symptoms(NMSs) treatment

Regarding medication selection for frequent NMSs, over 45.8% of responders employed Olanzapine rather than Clozapine (22.9%) for PD patients with psychosis. Memantine, donepezil, rivastigmine, and huperzine A were separately chosen by 37.8% (*n*=185), 53.8% (*n*=263), 16.8% (*n*=82), and 25.4% (*n*=124) of participants to improve CI in PD patients. Selective serotonin reuptake inhibitors (59.7%; *n*=292) were most popular agents for selecting to treat depression symptoms of PD patients. Only 20.7% and 37.8% of participants prefer to choose tricyclic antidepressants and pramipexole to deal with depression, respectively. Dopamine agonists, benzodiazepines and levodopa were selected by 30.5%, 25.2% and 28.6% of participants to deal with restless legs syndrome, respectively (Table 2).

There was a relatively high level of knowledge on therapeutic strategy among movement disorders specialists (Table 2) and those from tertiary hospitals and capital cities (Table 3). Doctors who had read the guidelines had significantly better knowledge of medication selections for PD under specific circumstances (Table 4).

**Table 3:** Drug selection strategy for PD patients under specific circumstances among doctors from different levels of hospitals and cities

**Items**	**Non-tertiary hospitals (*n*=147)**	**Tertiary hospitals (*n*=342)**	***P*value**	**Non-capital cities (*n*=259)**	**Capital cities (*n*=230)**	***P* value**
Age < 65 years without cognitive impairment						
Levodopa	60(40.8%)	104(30.4%)	0.025	102(39.4%)	62(27.0%)	0.004
Dopamine agonists	47(32.0%)	163(47.7%)	0.001	77(29.7%)	133(57.8%)	0.000
MAO-B inhibitors	15(10.2%)	47(13.7%)	0.282	22(8.5%)	40(17.4%)	0.003
Age > 65 years or with cognitive impairment						
Levodopa	86(58.5%)	191(55.8%)	0.587	127(49.0%)	150(65.2%)	0.000
Dopamine agonists	17(11.6%)	97(28.4%)	0.000	45(17.4%)	69(30.0%)	0.001
MAO-B inhibitors	12(8.2%)	29(8.5%)	0.908	19(7.3%)	22(9.6%)	0.375
Wearing-off phenomenon						
Add levodopa frequency	44(29.9%)	143(41.8%)	0.013	67(25.9%)	120(52.2%)	0.000
Switch to CR levodopa	67(45.6%)	169(49.4%)	0.436	106(40.9%)	130(56.5%)	0.001
Add COMT or MAO-B inhibitors	54(36.7%)	149(43.6%)	0.160	85(32.8%)	118(51.3%)	0.000
Add dopamine agonists	58(39.5%)	138(40.4%)	0.853	81(31.3%)	115(50.0%)	0.000
Peak-dose dyskinesia						
Reduce levodopa dose, add its frequency	55(37.4%)	163(47.7%)	0.037	91(35.1%)	127(55.2%)	0.000
Reduce levodopa dose, add dopamine agonists	58(39.5%)	157(45.9%)	0.188	97(37.5%)	118(51.3%)	0.002
Reduce levodopa dose, add COMT inhibitors	41(27.9%)	104(30.4%)	0.576	60(23.2%)	85(37.0%)	0.001
Add amantadine	17(11.6%)	54(15.8%)	0.224	31(12.0%)	40(17.4%)	0.089
PD with psychosis						
Clozapine	31(21.1%)	81(23.7%)	0.531	41(15.8%)	71(30.9%)	0.000
Olanzapine	59(40.1%)	165(48.2%)	0.099	98(37.8%)	126(54.8%)	0.000
Quetiapine	16(10.9%)	90(26.3%)	0.000	32(12.4%)	74(32.2%)	0.000
PD with dementia						
Huperzine A	51(34.7%)	73(21.3%)	0.002	53(20.5%)	71(30.9%)	0.008
Donepezil	70(47.6%)	193(56.4%)	0.073	126(48.6%)	137(59.6%)	0.016
Rivastigmine	15(10.2%)	67(19.6%)	0.011	41(15.8%)	41(17.8%)	0.555
Memantine	32(21.8%)	153(44.7%)	0.000	61(23.6%)	124(53.9%)	0.000
PD with depression						
Tricyclic antidepressants	41(27.9%)	60(17.5%)	0.010	65(25.1%)	36(15.7%)	0.010
SSRIs	86(58.5%)	206(60.2%)	0.721	144(55.6%)	148(64.3%)	0.049
Pramipexole	40(27.2%)	145(42.4%)	0.001	62(23.9%)	123(53.5%)	0.000
PD with RLS						
Levodopa	31(21.1%)	92(26.9%)	0.174	48(18.5%)	75(32.6%)	0.000
Dopamine agonists	31(21.1%)	118(34.5%)	0.003	56(21.6%)	93(40.4%)	0.000
Benzodiazepines	43(29.3%)	97(28.4%)	0.842	88(34.0%)	52(22.6%)	0.006

**Table 4:** The impact of PD guideline awareness on drug selection strategy under specific circumstances

**Items**	**Haven’t read the PD guideline(s) (*n*=97)**	**Have read the PD guideline(s) (*n*=392)**	***P*value**
Age < 65 years without cognitive impairment			
Levodopa	44(45.4%)	120(30.6%)	0.006
Dopamine agonists	25(25.8%)	185(47.2%)	0.000
MAO-B inhibitors	7(7.2%)	55(14.0%)	0.071
Age > 65 years or with cognitive impairment			
Levodopa	58(59.8%)	219(55.9%)	0.485
Dopamine agonists	17(17.5%)	97(24.7%)	0.132
MAO-B inhibitors	7(7.2%)	34(8.7%)	0.643
Wearing-off phenomenon			
Add levodopa dose	30(30.9%)	157(40.1%)	0.098
Switch from standard levodopa to CR levodopa	31(32.0%)	205(52.3%)	0.000
Add COMT inhibitors or MAO-B inhibitors	21(21.6%)	182(46.4%)	0.000
Add dopamine agonists	20(20.6%)	176(44.9%)	0.000
Peak-dose dyskinesia			
Reduce levodopa dose, add its frequency	41(42.3% )	177(45.2%)	0.609
Reduce levodopa dose, add dopamine agonists	31(32.0% )	184(46.9%)	0.008
Reduce levodopa dose, add COMT inhibitors	14(14.4%)	131(33.4%)	0.000
Add amantadine	9(9.3%)	62(15.8%)	0.102
PD with psychosis			
Clozapine	17(17.5%)	95(24.2%)	0.159
Olanzapine	42(43.3%)	182(46.4%)	0.580
Quetiapine	14(14.4%)	92(23.5%)	0.053
PD with dementia			
Huperzine A	26(26.8%)	98(25.0%)	0.715
Donepezil	51(52.6%)	212(54.1%)	0.790
Rivastigmine	8(8.2%)	74(18.9%)	0.012
Memantine	26(26.8%)	159(40.6%)	0.012
PD with depression			
Tricyclic antidepressants	28(28.9%)	73(18.6%)	0.026
SSRIs	45(46.4%)	247(63.0%)	0.003
Pramipexole	20(20.6%)	165(42.1%)	0.000
PD with RLS			
Levodopa	19(19.6%)	104(26.5%)	0.158
Dopamine agonists	20(20.6%)	129(32.9%)	0.019
Benzodiazepines	29(29.9%)	111(28.3%)	0.758

## Discussion

This survey summarized general recognitions and opinions on the diagnosis and treatment of PD since the publication and dissemination of the first edition of Chinese PD diagnosis criteria and treatment guideline in China in 2006. With global populations aging, there are more than two million PD patients in China by rough estimation, accounting for 40% PD patients in the world, which brings heavy economic burden to the communities and families [16]. Managing the large population of PD patients in clinical practice and getting more and more updated information from the movement disorders specialists in the world, Chinese doctors have significantly improved their ability to make diagnosis and carry out reasonable therapy for PD over the past three decades. However, rare reports focus on medications selection strategy among professional levels of doctors in China [17].

As disclosed in this survey, in contrast to the Western Countries, where only the movement disorders specialists have the qualifications to provide consultations to the PD patients referred from other doctors, both general neurologists and physicians are caring for PD patients in China, in addition to movement disorders specialists. In fact, in China neither the numbers of movement disorders clinics nor the movement disorders specialists are sufficient to cope with the increasing PD patients. These differences in our survey among doctors at different levels for PD clinics may reflect at least two aspects: one is a relative shortage of medical professional human resources of movement disorders specialists in China, and the second is that the corresponding subspecialty fellowship training programs are lacking [14,15,18].

From the perspective of doctors, this survey displays the general features on PD in China: most of the participants have adopted the Chinese PD guideline to instruct the clinical practice of PD; Whereas, CME activity frequency are very low and few of those surveyed have published PD related articles. The phenomenon reflected marked shortcomings of academic summary in routine clinical works among most of the surveyed, especially general neurologists and physicians. These pitfalls partly explained the reasons for shortage of influential multi-center clinical studies and original basic research for PD in China compared to Western countries and Japan.

With respect to diagnostic methods for PD, standard oral levodopa test and conventional MRI were commonly considered for suspected patients in routine practice, whereas SWI, TCS and functional neuroimaging were less referred. In China, relatively expensive functional neuroimaging methods, such as SPECT and PET are not covered by Chinese medical insurances, and few patients would like to choose them. Additionally, for loss of knowledge and shortage of TCS experts in many hospitals, TCS is rarely accepted as a regular method. During the past few years, accumulating cross-sectional and longitudinal studies across the globe indicated that several putative clinical symptoms, including hyposmia [19], RBD [20], constipation [21], depression [22], might assist the early diagnosis and differential diagnosis for PD. In this survey, nearly 40% of those participants would use aforementioned non-motor symptoms as assistant information in the diagnosis and differential diagnosis for PD in clinical practice. Meanwhile, the difference is significant among different stratified doctors, except anxiety and depression. More studies on pre-motor symptoms are warranted to compare the sensitivity and specificity of each non-motor symptom.

As expected, specialties (physicians, general neurologists and movement disorders specialists, Table 2), work places (the hospitals and the located cities, Table 3), and the Guideline awareness (Table 4) play important roles for reasonable PD medications selection strategy. There was a relatively high level of knowledge on therapeutic strategy under specific circumstance among movement disorders specialists and those from tertiary hospitals. However, the discrepancy between the guideline and clinical practice still exists on management of non-motor symptoms among all of those surveyed: Up to 45.8% of responders selected olanzapine to treat PD patients with psychosis, only 22.9% of those prescribed clozapine. The most often prescribed was donepezil, not rivastigmine, for PD with dementia, etc. Based on those observations, great efforts should be made to take the CME programs for physicians and general neurologists. Popularization activities of PD guideline across the country may be a visible strategy to obtain such purpose. In addition to establishment of clinical fellowship training system, the birth and development of movement disorders clinic team are necessary to standardize the diagnosis and treatment of PD. Interdisciplinary communication and cooperation among physicians, general neurologists and movement disorders specialists are warranted to optimize the management of PD, especially for late stage patients with PD.

However, there are some limitations in this survey. As an on-the-spot investigation, the questionnaire was distributed at various conferences and CME courses. There was a potential selection bias as these meetings attendants were likely more academic active compared to those absentees. Additionally, there are rare studies for cognition and attitude of expertise in the public health populations in China so far. Therefore, a series of investigations focused on the expertise and CME will be promoted on a large scale in the future.

## Conclusions

The national survey revealed that SWI complements MRI, TCS and functional neuroimaging were less performed for diagnosis of PD in clinical practice in China compared with commonly employed standard oral levodopa test and conventional MRI. The choices of diagnostic methods and therapeutic strategy of PD vary among physicians, general neurologists and movement disorders specialists. Guideline awareness is markedly beneficial to reasonable PD medications strategy in China. Further CME programs on the diagnosis and management of PD patients are warranted especially for physicians and general neurologists caring for PD patients in China.

## Abbreviations

PD: Parkinson’s disease; NMSs: Non-motor symptoms; CME: Continuing medical educations; SWI: Susceptibility weighed imaging; TCS: Transcranial sonography; SPECT: Single photon emission computed tomography; PET: Positron emission tomography; RBD: REM sleep behavior disorder; CI: Cognitive impairments.

## Competing interests

The authors declare that they have no competing interests.

## Authors’ contributions

*Study concept and design:* GW, WC, SDC. *Acquisition of data:* WC, SC, QX, GW, SDC. *Statistical analysis:* WC, SC. *Analysis and interpretation of data:* WC, GW, SDC. *Drafting of the manuscript:* WC. *Critical revision of the manuscript for important intellectual contents:* GW, SDC. *Study supervision:* QX, SDC. All authors read and approved the final manuscript.

## Pre-publication history

The pre-publication history for this paper can be accessed here:

http://www.biomedcentral.com/1471-2377/12/155/prepub

## Supplementary Material

Additional file 1 Clinical practice for Parkinson’s disease (PD): A questionnaire among Chinese doctors (English version). Click here for file

## References

[B1] Zhang ZX Roman GC Hong Z Parkinson's Disease in China: prevalence in Beijing, Xian, and Shanghai Lancet 2005 365 595 597 15708103 10.1016/S0140-6736(05)17909-4

[B2] Chen SD (The Chinese movement disorders and Parkinson’s disease society): The guideline for management of Parkinson’s disease in China. Chin J Neurol 2006 39 409 412

[B3] Chen SD (The Chinese movement disorders and Parkinson’s disease society): The guideline for management of Parkinson’s disease in China (second edition). Chin J Neurol 2009 42 352 355

[B4] Miyasaki JM Shannon K Voon V Practice parameter: evaluation and treatment of depression, psychosis, and dementia in Parkinson disease (an evidence-based review): report of the quality standards subcommittee of the American academy of neurology Neurology 2006 66 996 1002 10.1212/01.wnl.0000215428.46057.3d 16606910

[B5] Pahwa R Factor SA Lyons KE Practice parameter: treatment of Parkinson disease with motor fluctuations and dyskinesia (an evidence-based review): report of the quality standards subcommittee of the American academy of neurology Neurology 2006 66 983 995 10.1212/01.wnl.0000215250.82576.87 16606909

[B6] Suchowersky O Gronseth G Perlmutter J Reich S Zesiewicz T Weiner WJ Practice parameter: neuroprotective strategies and alternative therapies for Parkinson disease (an evidence-based review): report of the quality standards subcommittee of the American academy of neurology Neurology 2006 66 976 982 10.1212/01.wnl.0000206363.57955.1b 16606908

[B7] Suchowersky O Reich S Perlmutter J Zesiewicz T Gronseth G Weiner WJ Practice parameter: diagnosis and prognosis of new onset Parkinson disease (an evidence-based review): report of the quality standards subcommittee of the American academy of neurology Neurology 2006 66 968 975 10.1212/01.wnl.0000215437.80053.d0 16606907

[B8] National Collaborating Centre for Chronic Conditions (UK) Parkinson’s Disease: National clinical guideline for diagnosis and management in primary and secondary care [Internet] 2006 Available from URL: http://www.nice.org.uk/CG035. 21089238

[B9] Tian YY Tang CJ Wu J Zhou JS Parkinson's Disease in China Neurol Sci 2011 32 23 30 21174138 10.1007/s10072-010-0461-8

[B10] Fargel M Grobe B Oesterle E Hastedt C Rupp M Treatment of Parkinson's disease: a survey of patients and neurologists Clin Drug Investig 2007 27 207 218 10.2165/00044011-200727030-00004 17305415

[B11] Eggert K Larisch A Dodel R Bormann C Oertel WH Awareness and knowledge of the clinical practice guideline on Parkinson's disease among German neurologists Eur Neurol 2009 61 216 222 10.1159/000197106 19176962

[B12] Larisch A Oertel WH Eggert K Attitudes and barriers to clinical practice guidelines in general and to the guideline on Parkinson's disease. A National Survey of German neurologists in private practice J Neurol 2009 256 1681 1688 10.1007/s00415-009-5178-3 19479167

[B13] Willis AW Schootman M Evanoff BA Perlmutter JS Racette BA Neurologist care in Parkinson disease: a utilization, outcomes, and survival study Neurology 2011 77 851 857 10.1212/WNL.0b013e31822c9123 21832214 PMC3162639

[B14] Guo Y Shibuya K Cheng G Rao K Lee L Tang S Tracking China's health reform Lancet 2010 375 1056 1058 10.1016/S0140-6736(10)60397-2 20346797

[B15] Shi FD Jia JP Neurology and neurologic practice in China Neurology 2011 77 1986 1992 10.1212/WNL.0b013e31823a0ed3 22123780 PMC3235353

[B16] Wang G Cheng Q Zheng R Economic burden of Parkinson's disease in a developing country: a retrospective cost analysis in Shanghai, China. Mov Disord 2006 21 1439 1443 10.1002/mds.20999 16773620

[B17] Wang G Zhou HY Zheng R Investigation on the clinical use of anti-Parkinson’s disease drugs for patients with Parkinson’s disease J Clin Neurol 2006 19 336 338

[B18] Wang XP Zhang WF Huang HY Preter M Neurology in the People's republic of China–an update Eur Neurol 2010 64 320 324 10.1159/000321648 21071947

[B19] Ross GW Petrovitch H Abbott RD Association of olfactory dysfunction with risk for future Parkinson's disease Ann Neurol 2008 63 167 173 10.1002/ana.21291 18067173

[B20] Iranzo A Valldeoriola F Lomena F Serial dopamine transporter imaging of nigrostriatal function in patients with idiopathic rapid-eye-movement sleep behaviour disorder: a prospective study Lancet Neurol 2011 10 797 805 10.1016/S1474-4422(11)70152-1 21802993

[B21] Abbott RD Petrovitch H White LR Frequency of bowel movements and the future risk of Parkinson's disease Neurology 2001 57 456 462 10.1212/WNL.57.3.456 11502913

[B22] Walter U Hoeppner J Prudente-Morrissey L Horowski S Herpertz SC Benecke R Parkinson's Disease-like midbrain sonography abnormalities are frequent in depressive disorders Brain 2007 130 1799 1807 10.1093/brain/awm017 17329323

