# Supplementary material for: Current clinical practice for Parkinson’s disease among Chinese physicians, general neurologists and movement disorders specialists: a national survey
Source: BMC Neurol. 2012 Dec 7;12:155. doi: 10.1186/1471-2377-12-155 (PMC3538053; doi:10.1186/1471-2377-12-155)
Supplement: Additional file 1 — Clinical practice for Parkinson’s disease (PD): A questionnaire among Chinese doctors (English version). [file 1471-2377-12-155-S1.doc]

Number: ______________

**Clinical practice for Parkinson’s disease (PD): A questionnaire among Chinese doctors**

(The authors translated this questionnaire from its original Chinese version)

**Ⅰ. Demographic information**

- Sex ①male ②female
- Age ①≤35years old ②35-60years old ③≥60 years old
- Address Province _________ City____________
- Hospital ①Private clinic or primary health center ②Secondary class hospital ③Tertiary class hospital
- Specialty ①Physician ②General neurologist ③Movement disorders specialist
- Professional qualification ①Resident ②Attending ③Vice chief physician ④ Chief physician

**Please select one or multiple answers for each question below according to your recent experiences and expertise.**

**Ⅱ. Continuing Medical Educations(CME) & research experiences for PD**

- How do you keep your diagnosis and treatment of PD up to date? Via ①Movement disorders textbooks ②Information from the pharmaceutical sales representatives ③Lectures of movement disorders specialists ④Professional journals ⑤Diagnosis & treatment Guidelines
- Whether have you read the following guidelines for PD? ①AAN guidelines in the USA ②NICE guideline in UK ③EFNS in Europe ④First version of Chinese PD guideline(2006) ⑤Updated version of Chinese PD guideline(2009) ⑥None of them above
- How often have you attended CME activities on PD and related disorders? ①More than 5 times each year ②3-5 times each year ③1-2 times each year ④Never
- How many PD patients(including new and follow-up ones) have been seen by you per month? ①None ②Less than 10 ③10-30 ④More than 30
- Have you published research papers on PD and related disorders in professional periodicals? ①Yes, both clinically and basically ②Yes, clinically ③Yes, basically ④No
- Are you undertaking basic research on PD? ①Yes ②No

**Ⅲ. Initial diagnostic approaches for PD**

- Would you like to try a standard oral levodopa test for suspected PD patients? ①Yes ②No
- Whether will you select the following imaging methods to support you diagnosis? ①MRI ②PET ③SPECT ④None
- Which cranial magnetic resonance imaging sequence would you like to choose for PD patients? ①T1WI ②T2WI ③DWI ④FLAIR ⑤SWI
- Have you performed transcranial sonography (TCS) for PD patients? ①Never heard of ②Haven’t started, but have a plan ③Already started
- Which non-motor symptoms do you think are the putative pre-motor biomarkers? ①Hyposmia ②Rapid eye movement sleep behavior disorders ③Constipation ④Anxiety & depression ⑤Cognitive impairment ⑥Psychosis

**Ⅳ. Strategy on PD management**

- What’s your initial medication for newly diagnosed PD patients with age below 65 years and without cognitive impairment? ①Levodopa ②Dopamine agonists ③Amantadine ④Benzhexol ⑤MAO-B inhibitors ⑥ COMT inhibitors
- What’s your initial medication for newly diagnosed PD patients with age above 65 years or with cognitive impairment? ①Levodopa ②Dopamine agonists ③Amantadine ④Benzhexol ⑤MAO-B inhibitors ⑥ COMT inhibitors
- What’s your common adjustment strategy for treatment of PD patients with “wearing-off” phenomenon? ①Add levodopa frequency②Switch to CR levodopa ③Add COMT inhibitors or MAO-B inhibitors ④Add dopamine agonists
- What’s your common adjustment strategy for treatment of PD patients with peak dose dyskinesia? ①Reduce levodopa dose, add its frequency②Reduce levodopa dose, add dopamine agonists ③Reduce levodopa dose, add COMT inhibitors ④Add amantadine
- Which antipsychotics would you choose for patients with visual hallucination or delusion after failure of adjusting related anti-PD drugs? ①Clozapine ②Olanzapine ③Quetiapine ④Benzodiazepines
- Which drug would you choose to improve cognitive impairment for patients with dementia(PDD)? ①Huperzine A ②Donepezil ③Rivastigmine ④Memantine ⑤ Others

| - Which drug would you choose for PD patients with depression? ①Tricyclic antidepressants ②Selective serotonin reuptake inhibitors(SSRIs) ③Pramipexole ④[Serotonin noradrenaline reuptake inhibitors(](http://www.baidu.com/link?url=b58b924dea32265d136df622f496a4d9f6a3cf872c05adaa3bc6c184a18c4c353204a2e068cfb9dd65f1288671e08d115bac051fea2098bebf56b4ef094450528b2921d781eae0a0f9194afed4fcdc0c355040412bd0c50cba6e465beabac86bca4255615259c9d6f0199cec855a7eda76bc2bb08bea8a3d15064d9845ee52ecf83eac1b25d84030af18a0a3778095ebb08ba25fed4a3aacfe6a6354373dbf3ff3438f2b8aed033b09da1daa9ce9f3c012b45a4ac0fb2254deee6fa941fb69fa959e2c0f38db4ab615e2891549892d2a694f0fbdefb2bc83db54c42334599feaf0997af96e99c01d255c91f741ee6d8915c68a976468fbd83d2d46d6db18ab9a91594cfdabc380effd2dcd16ac26c32836d4824ae3f5fdb838e874f12a0f1779bcfe5dcc834e938ec64e5ed4bbfd105e8d73d1d9a5e4a3bc971193b41da017742e346408597e640ff53d4b2a1d543cc1161789113fa6d885ac319f33b557ebac5573358cc3c0659257bfcf546427d385eaf17664ea5e0064419878f61d6a7da437f769b038d3ff55f00ea01c63)SNRIs) ⑤ Traditional Chinese Medicine |
| --- |

- Which drug would you choose for PD patients with Restless legs syndromes? ①Levodopa ②Non-ergot dopamine agonists ③Amantadine ④Benzhexol ⑤Benzodiazepines
